# Genome-wide analyses of *Mycobacterium tuberculosis* complex isolates reveal insights into circulating lineages and drug resistance mutations in The Gambia

**DOI:** 10.1038/s41598-026-42003-2

**Published:** 2026-03-04

**Authors:** Fatou Faal, Naffie Top, Olimatou Jobe, Sang M. Colley, Abigail Ayorinde, Alieu Mendy, Binta Sarr-Kuyateh, Simon Donkor, Martin Antonio, Bouke C. de Jong, Andrea Rachow, Beate Kampmann, Jayne S. Sutherland, Hongwei Li, Tom L. Blundell, Susana Campino, Thomas Kohl, Viola Dreyer, Stefan Neimann, Arun P. Pandurangan, Taane G. Clark, Jody E. Phelan, Leopold D. Tientcheu

**Affiliations:** 1 MRC Unit The Gambia at London School of Hygiene and Tropical Medicine, Banjul, The Gambia; 2 Department of Infection Biology, Faculty of Infectious and Tropical Diseases, London School of Hygiene and Tropical Medicine, London, UK; 3 Institute of Tropical Medicine, Antwerp, Belgium; 4 Institute of Infectious Diseases and Tropical Medicine, LMU University Hospital, LMU Munich, Munich, Germany; 5 German Center for Infection Research, Partner site Munich, Munich, Germany; 6 Unit Global Health, Helmholtz Zentrum München, German Research Centre for Environmental Health (HMGU), Neuherberg, Germany; 7 Charité – Universitätsmedizin Berlin, Berlin, Germany; 8 VPD Heart and Lung Research Institute, University of Cambridge, Cambridge, UK; 9 Molecular and Experimental Mycobacteriology, Research Center Borstel, Parkallee 1, 23845 Borstel, Germany; 10 Gonville and Caius College, University of Cambridge, Cambridge, UK; 11 Faculty of Epidemiology and Population Health, London School of Hygiene and Tropical Medicine, London, UK; 12 Department of Biochemistry, Faculty of Sciences, University of Yaoundé 1, Yaoundé, Cameroon

**Keywords:** Computational biology and bioinformatics, Genome informatics, Microbiology, Microbial genetics, Bacterial genes

## Abstract

Tuberculosis (TB), caused by the *Mycobacterium tuberculosis* complex (MTBC), remains a pressing global health challenge, with a high burden in West Africa, including The Gambia. Understanding the genetic diversity of circulating MTBC strains is essential for improving diagnosis, surveillance and treatment strategies. In this study, we characterise the population structure and drug resistance landscape of MTBC strains circulating in The Gambia over nearly two decades (2002–2021). We analysed whole-genome sequencing (WGS) data from 1,803 TB isolates. Lineage 4 (L4) was predominant (67.2%), followed by the West Africa-restricted lineage 6 (L6, 26.6%), with L4 exhibiting greater genetic diversification over time. Drug susceptibility profiling of these isolates revealed that 78% (1421/1803) were drug-susceptible, while 6.5% (119/1803) harboured resistance to first-line drugs, primarily to isoniazid, rifampicin, or both. Notably, 15.5% (282/1803) isolates carried mutations classified as having uncertain significance according to the WHO resistance catalogue. Comparative analyses revealed a lineage 6-specific ethambutol-associated mutation of uncertain significance (*em*bC Ala307Thr) occurring at a higher frequency in Gambian isolates than in the broader West Africa region or globally. Structural modelling demonstrated that many first-line drug resistance mutations are located in highly conserved, solvent-inaccessible regions of target proteins, often impacting protein stability, suggesting a trade-off between drug resistance, bacterial fitness, and evolutionary adaptation. Together, these findings highlight the coexistence of globally widespread and regionally restricted MTBC lineages in The Gambia and reveal a substantial burden of resistance-associated mutations of uncertain significance in the WHO catalogue. Sustained genomic surveillance and region-specific interpretation of resistance mutations are essential to support End TB strategies in high-burden settings.

**Supplementary Information:**

The online version contains supplementary material available at 10.1038/s41598-026-42003-2.

## Introduction

Tuberculosis (TB) caused by bacilli of the *Mycobacterium tuberculosis* complex (MTBC) remains a significant public health problem worldwide. In 2022, an estimated 10.6 million people developed TB, with 1.3 million deaths reported. West Africa accounted for 10% of global TB deaths in 2022, including 3,900 cases and 580 fatalities in The Gambia. TB disproportionately affects resource-limited communities and low-income countries^1^.

Whole genome sequencing (WGS) advances have greatly enhanced our understanding of MTBC lineage diversity and phylogeographical distribution^2,3^. Of the ten known MTBC lineages (L1-L10)^4^, all are found in Africa. In West Africa, TB is driven by *M. tuberculosis* sensu stricto (*Mtb*) and *M. africanum (Maf)* lineages, which co-exist in affected populations^5^. While geographically restricted lineages like *M. africanum* (L5, L6) are endemic to West Africa, globally disseminated lineages such as *Mtb*-Beijing (L2) and *Mtb*-Europe-America (L4) are also prevalent^6^.

West Africa follows the World Health Organisation (WHO) guidelines for TB treatment, involving a prolonged multi-drug regimen^1^. TB patients’ treatment encompasses two regimens: drug-susceptible (DS) and drug-resistant (DR). DS-TB patients undergo a six-month treatment course comprising an intensive phase with rifampicin (RIF), isoniazid (INH), ethambutol (EMB), and pyrazinamide (PZA) for two months, followed by a four-month continuation phase with RIF and INH^7^. For DR-TB cases, treatment regimens are adjusted based on resistance profiles, often requiring second-line drugs and extended treatment durations, which complicate patient management and result in poorer treatment outcomes^8,9^.

Previous studies have shown that TB patient responses to standard anti-TB treatment vary depending on the infecting MTBC lineages, notably their immune response^10–14^. Moreover, hypervirulent strains can dampen immune defences, leading to accelerated disease progression and increased transmission rates^15^.

The mutation rates of lineages have been found to vary significantly, with some lineages exhibiting a higher propensity for developing mutations that confer resistance to primary TB drugs^16^. For example, L2 has been noted for its increased mutation rates, particularly in drug-resistant (DR) strains, contributing to intrinsic treatment challenges and the emergence of multidrug-resistant (MDR) TB^17–19^. The persistence and proliferation of resistant strains during treatment can lead to therapeutic failures and complicate TB control efforts^20^.

This study investigates the genetic diversity of MTBC strains circulating in The Gambia over nearly two decades (2002 to 2021) and explores their implications for effective TB management. By integrating structural bioinformatics and computational approaches, we analysed the most extensive WGS collection of MTBC isolates in a West African country. We present the abundance, distribution and effects of genetic mutations on drug-target protein stability and conservation properties. Understanding these genetic variations is crucial for designing new drugs and developing effective TB treatment strategies, particularly in regions like West Africa, where multiple MTBC lineages coexist.

## Methodology

### Ethical statement

This was a retrospective study that received ethical approval from the Gambian Government/MRC joint Ethics Committee and the London School of Hygiene and Tropical Medicine Ethics Committee. All the previous studies we used data from were ethically approved by the Gambian Government/MRC joint Ethics Committee, and written informed consent was obtained from either the participant or their guardian. All laboratory procedures and data handling were conducted in accordance with established ethical guidelines to ensure confidentiality.

### Sequencing and epidemiological data

The whole genome sequences (WGS) and epidemiological data used in this analysis (*n* = 1803) were sourced from consecutive TB projects hosted by the TB case contact platform at the MRCG@LSHTM between 2002 and 2021. These projects include the following with their respective number of WGS samples: PRJEB53138 (Enhance Case Finding; *n* = 1302), SCC 1289 (Childhood TB Program; *n* = 234), SCC 1523 (TB Sequel; *n* = 216) and Recurrent TB (*n* = 52). For each isolate, accompanying demographic and clinical metadata, including participant age, sex, and year of sample collection, were extracted and included in the analysis.

### Microbiology and DNA extraction

Briefly, stored MTBC isolates from archived stocks or directly from a microbiology growth indicator (MGIT^™^) positive tube were subcultured into Middlebrook 7H9 broth or Lowenstein-Jensen (LJ) slopes to multiply the colonies. Genomic DNA was extracted using the cetyltrimethylammonium bromide (CTAB) method, as previously described^21^. The extracted DNA underwent WGS on the Illumina HiSeqX platform at the Forschungszentrum Research Center Borstel, Germany. For the PRJEB53138 isolates, the DNA was extracted using Maxwell^®^ 16 Viral Total Nucleic Acid Purification Kit (Promega Corporation, Fitchburg, WI, USA) following the manufacturer’s instructions and sequenced in MicrobsNG in the United Kingdom.

### Bioinformatic and phylogenetic analysis

Raw sequence data (approximately 2000 samples) was processed and analysed to ensure data quality and accuracy. The Kraken2 database tool was used to filter contaminated sequences and exclude non-MTBC strains. Poor-quality reads were trimmed using Trimmomatic (v0.39) with the following parameters: LEADING:3 TRAILING:3 SLIDINGWINDOW:4:20 MINLEN:36. The quality of the processed reads was reassessed using FastQC.

For each sample, trimmed reads were aligned to the *Mycobacterium tuberculosis* H37Rv reference genome (accession: NC_000962.3) using BWA-MEM software^22^. Single nucleotide polymorphisms (SNPs) and insertions/deletions (indels) were identified through the application of the Genome Analysis Toolkit (GATK)^23^ and Sequence Alignment Map (SAM)^24^ tools. Genomic VCF files from all the samples were merged, and multi-FASTA alignments were generated using BEDTools software.

Phylogenetic relationships between the samples were inferred by constructing a phylogenetic tree with IQ-TREE software^25^. The tree was visualised and annotated using the Interactive Tree of Life (iTOL) v6 software platform^26^. The tree was visualised and annotated using the iTOL software platform. MTBC lineages and genotypic drug resistance profiles for each isolate were determined using the TB-Profiler pipeline (v4.4.0; database version: e25540b)^27^. Variants, including missense and frameshift mutations within known drug resistance loci, were analysed and compared to established databases such as TB-Profiler and the WHO catalogue to identify reported and potential unreported polymorphisms. Mutations in Tier1 genes for the Gambian dataset were compared with global mutation data (> 100 K mutations) and specific datasets from other West African countries^28,29^.

The analysis included detailed information for each mutation, including the gene names, nucleotide changes, mutation frequency, and count for each country, identified by their country codes. This comprehensive approach provided insight into regional and global genetic diversity and drug resistance dynamics in MTBC strains.

### Protein structural modelling and mutant stability prediction

Missense mutations associated with first-line drugs (RIF, INH, PZA, and EMB) were filtered using a frequency cutoff of 80% to ensure accuracy and relevance. Redundant mutations within the same gene were identified and removed to clarify the dataset and eliminate duplication. The final dataset comprised 943 isolates and their associated missense mutations, of which 614 were classified as susceptible and 329 as resistant. This curated dataset offers a comprehensive overview of the genetic basis of drug susceptibility and resistance.

Protein structures for the target genes were obtained from the Protein Data Bank (PDB), a key repository of experimentally determined macromolecular structures critical for biomedical research and drug discovery^30,31^. This dataset included four PDB files derived from experimentally determined crystal structures and twenty predicted structures using AlphaFold, a protein structure prediction tool^32^. To predict the stability changes caused by mutations, the Delta Delta G (ΔΔG), representing the difference in free energy between wild-type and mutant protein forms, was calculated using PyRosetta, FoldX, and site-directed mutator (SDM) (https://compbio.medschl.cam.ac.uk/sdm2/)^33–35^.

ConSurf (https://consurf.tau.ac.il/consurf_index.php) was employed to evaluate the evolutionary conservation of amino acids at mutation sites. Conservation grades, ranging from 1 (highly variable) to 9 (highly conserved), were assigned based on the evolutionary significance of specific residues^36^. Highly conserved residues are often critical for structural integrity or functional roles in proteins. The conservation grades for mutation positions were extracted and compared between mutations classified as resistant and susceptible. This comparison offered insights into the mutations’ evolutionary importance and potential functional consequences.

## Results

### Study demography

This study analysed 1803 MTBC isolates with whole-genome sequencing (WGS) data from TB patients residing in the Greater Banjul Area, which accounts for 80% of all TB cases in The Gambia. Metadata, including either age, sex, or year of sample collection, was available for 1713 isolates (95%) (Table 1). The majority of isolates (1145/1585, 72.2%) were from male patients, with the highest representation among individuals aged 18–29 (498, 32%) and 30–44 (409, 26.3%). Most samples were from patients diagnosed between 2012 and 2015 (1433/1713, 83.6%) (Supp Fig. 1).

**Table 1:** Characteristics of the MTBC strains and underlying patients (*N* = 1803).

Characteristics	Number of isolates (*N*)	Percentage (%)
Year of diagnosis		
2002–2011	38	2.11
2012	338	18.75
2013	471	26.12
2014	585	32.45
2015	39	2.16
2016	26	1.44
2017	23	1.28
2018	94	5.21
2019	93	5.16
2020	6	0.33
NA	90	4.99
Age groups(yrs)		
Under 18	18	0.9
18–29	696	38.6
30–44	551	30.5
45 and above	287	15.9
NA	251	13.9
Sex		
Female	440	24.4
Male	1145	63.5
NA	218	12
Lineage		
L4	1214	67.2
L6	480	26.6
L2	59	3.2
L1	25	1.3
L3	21	1.1
L5	4	0.2
Genotypic drug res		
Pan sensitive	1421	78
RIF	10	0.5
INH	90	5
MDR	19	1
Other	282	15.5

RIF = Rifampicin, INH = Isoniazid, MDR = multi-drug resistance, Other= Known drug resistance mutation but classified as uncertain significant by the WHO catalogue.

**Fig. 1:**
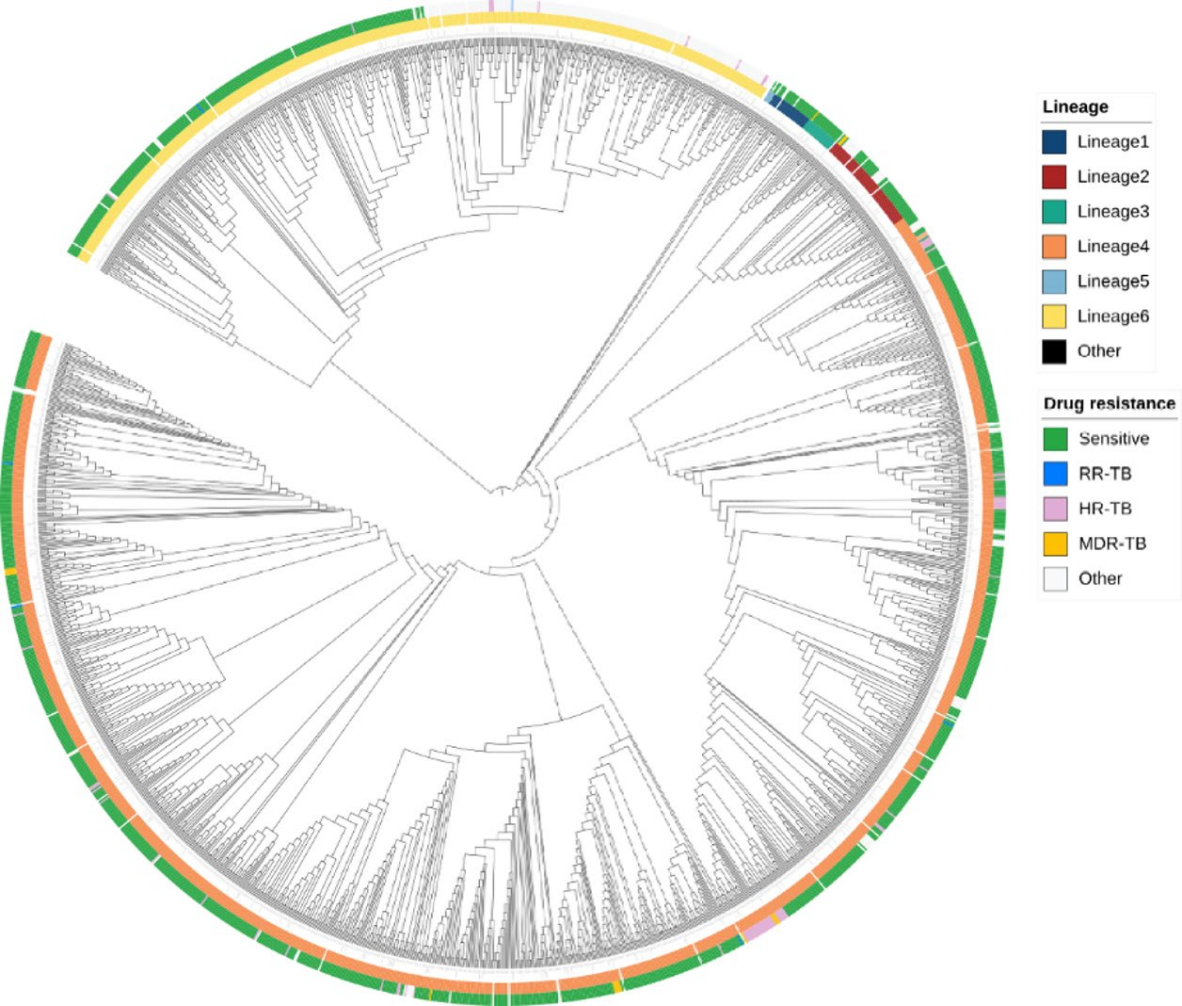
Phylogenetic relationship and drug resistance profile of 1803 MTBC isolates. The outer circle represents drug susceptibility output based on genotyping: drug-sensitive isolates (Green), Rifampicin mono-resistant (RR-TB; blue), Isoniazid mono-resistant (HR-TB; soft magenta), multi-drug-resistant (MDR; Yellow), and isolates resistant to other drugs (Other; White). The inner circle consists of MTBC lineages: Lineage 1 (Dark blue), Lineage 2 (Brown), Lineage 3 (Green), Lineage 4 (Orange), Lineage 5 (Light blue), Lineage 6 (Yellow) and Other (Black). The branches represent a clustering of isolates based on SNP differences.

### MTBC lineages

Phylogenetic analysis revealed the clustering of isolates by lineage, confirming the dominance of specific MTBC lineages in the population (Fig. 1). Most isolates (94%) belonged to *Mtb* Lineage 4 (L4; 1214/1803, 67.2%) and *Maf* Lineage 6 (L6; 480/1803, 26.6%) (Table 1). L4 has remained the predominant lineage throughout the study period (Supp Fig. 1). Among the sub-lineages, L4.1 (410 isolates) and L4.3 (LAM; 323 isolates) were the most abundant within L4, while L6.1 was the most common sub-lineage of L6 (Supp Fig. 2).

**Fig. 2:**
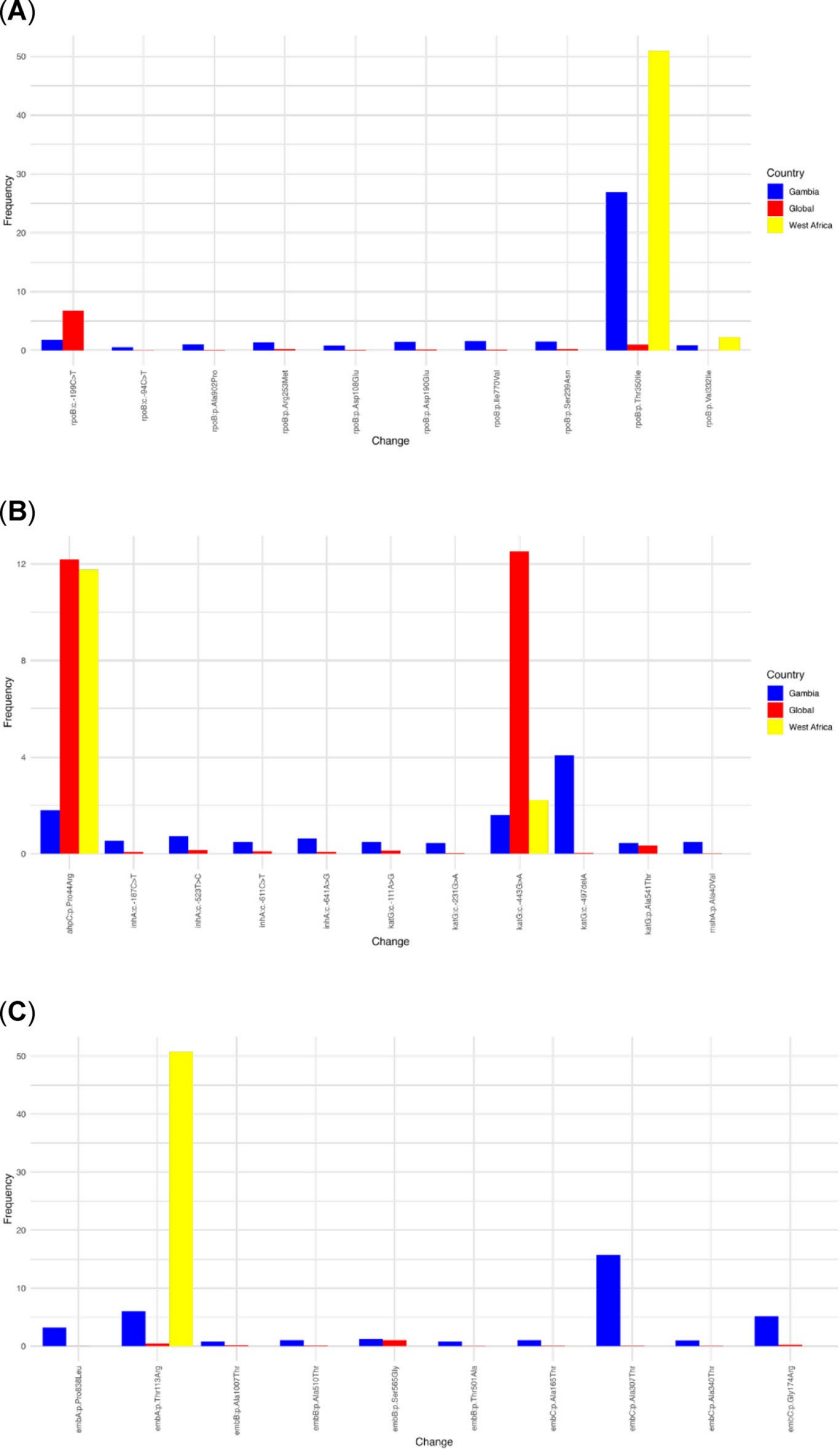

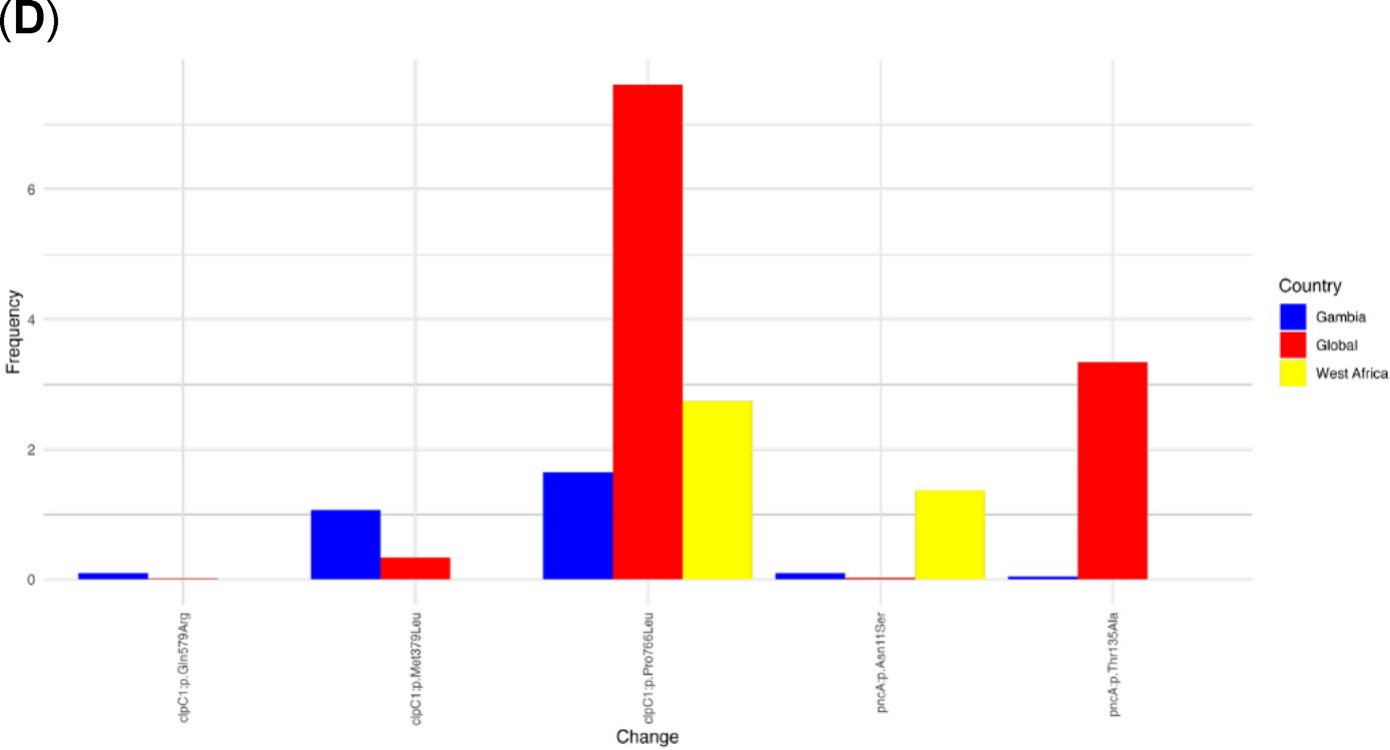
Diversity and prevalence of the WHO catalogue’s mutations, with uncertain significant resistance to the first-line drug-targeted genes across The Gambia, West Africa, and the global dataset. Figure panels A to D show the mutation for each of the first-line anti-TB drugs. In each figure, the Y-axis shows the frequency of each gene mutation displayed on the X-axis in the three regions of interest: The Gambia (Blue), West Africa (Yellow), and the rest of the globe (Red). Panel (**A**) represent gene mutation associated with Rifampicin, panel (**B**) Isoniazid, panel (**C**) Ethambutol, and panel (**D**) Pyrazinamide.

### Drug resistance

The isolates’ genotypic resistance profile revealed that 1421 (78%) were drug-susceptible (DS). Among the drug-resistant (DR) isolates, 90 (5.0%) were resistant to INH alone, 10 (0.6%) were resistant to RIF alone, and 19 (1.1%) were multidrug-resistant (MDR). The WGS analysis revealed the following distribution of MDR isolates: 1 case in 2002, 2003, 2008, 2012, 2013, 2018 and in an unknown year of collection. Whereas, 8 cases were detected in 2014 and 2 in 2015 (Suppl. Figure 3B). Additionally, 282 isolates (15.3%) were classified as other, having potential drug-resistance associated mutations, identified by the TB-Profiler pipeline^27^. The most frequent mutation underlying resistance to INH was *kat*G Ser315Thr, observed in 71 isolates (71/90, 78.8%) (Table 2). Lineage-specific mutations were also detected in known drug-resistance genes. For instance, *emb*C Ala307Thr, a mutation associated with ethambutol but classified as uncertain significant by the WHO catalogue, was specific to L6, with a frequency of 56.34% (270/480). Another ethambutol-associated mutation of uncertain significance, *emb*A Thr113Arg, was also unique to L6 with a frequency of 22.61% (106/480) (Table 2). According to the drug resistance classification, INH resistance (HR-TB) and MDR-TB were predominantly observed in lineage 4. In contrast, lineage 6 harboured the highest number of mutations classified as “Other,” including mutations of uncertain significance defined by the WHO catalogue. INH and these “Other” mutations were mainly detected between 2012 and 2019 (Supp Fig. 3).

**Fig. 3:**
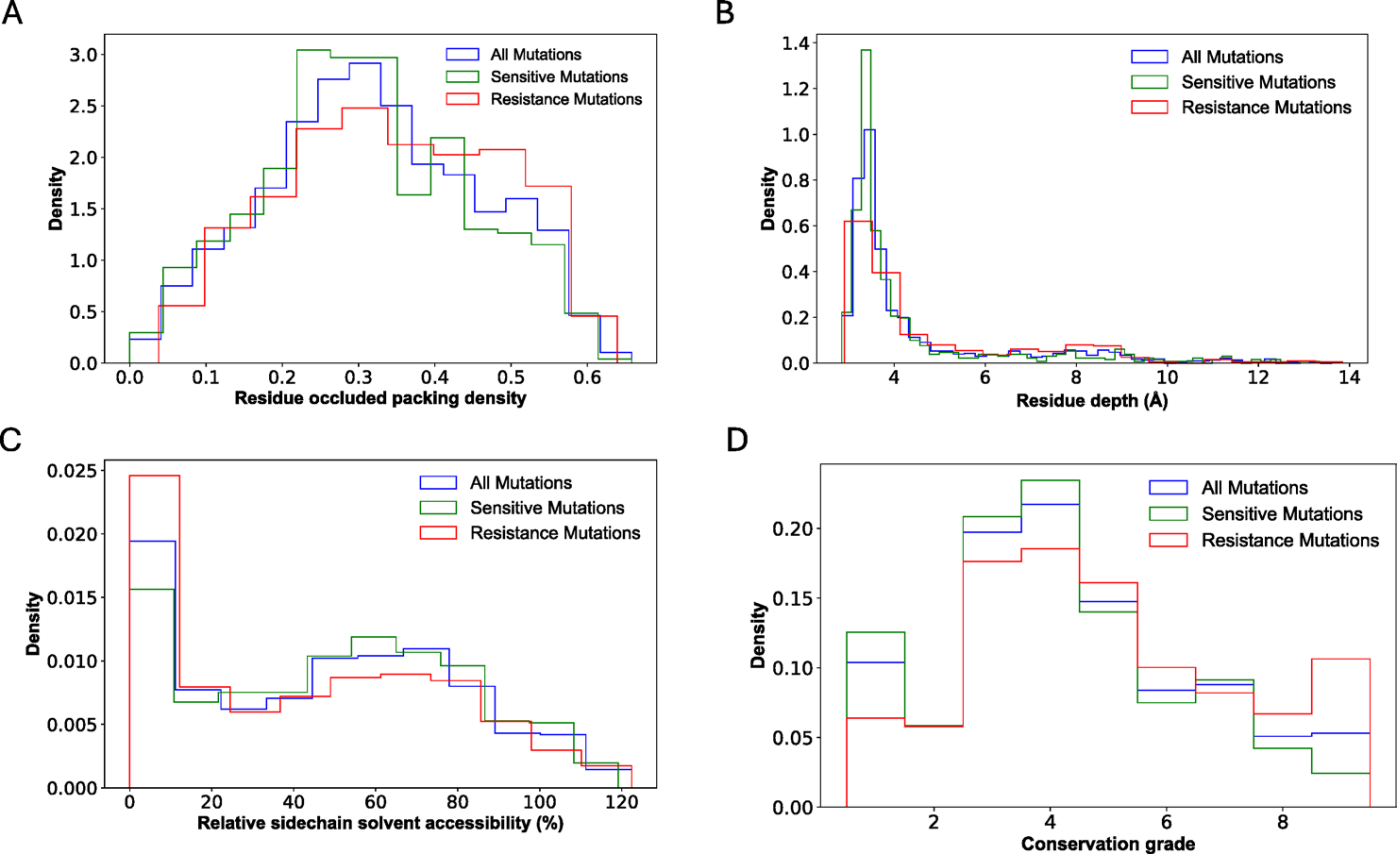
Structural and conservation properties for resistant, susceptible and background mutations. Panel (**A**) Residue occluded packing density, (**B**) Residue depth, (**C**) Relative side chain solvent accessibility and (**D**) Residue conservation. The line pattern shows the density of each mutation type for the different parameters evaluated. Blue line represents the background mutations (occurring in both resistant and susceptible isolates), Green line represents sensitive mutations (occurring only in drug-susceptible isolates), and Red line represents resistance mutations (occurring in drug-resistant isolates).

**Table 2:** Mutations with known and unknown resistance significance in first-line drugs.

Drug	Gene	Mutation	Frequencies in The Gambia (%)	West-African frequencies (%)	Global frequencies (%)	Lineage (L)
Rifampicin	*rpo*B	Thr350Ile	26.9	50.9	0.99	6
		c.−199 C > T	1.79	NA	6.75	4
		Ser450Leu	0.44	28.4	34.5	2,3,4
		Asp435Val	0.44	7.43	3.01	4
		Ile770Val	1.6	NA	0.11	4,6
Isoniazid	*kat*G	Ser315Thr	3.84	48.34	39.34	2,3,4,6
		c.497delA	4.07	NA	0.02	4
	*inh*A	c.−777 C > T	0.72	6.32	13.33	1,2,3,4,5,6
		c.−154G > A	0.33	9.23	1.86	4,6
	*ahp*C	Pro44Arg	1.79	11.7	12.17	4
Ethambutol	*emb*A	Thr113Arg	6.02	50.74	0.45	6
	*emb*C	Ala307Thr	15.73	NA	0.05	6
	*emb*B	Met306Val	0.19	3.66	13.85	2,4
		Met306Ile	0.14	7.02	12.32	4
Pyrazinamide	*clp*C1	Pro766Leu	1.65	2.74	7.60	4
	*pnc*A	Leu172Pro	0.19	2.22	0.61	1,2,3,4,5
		Asp63Ala	0.14	NA	0.97	2,3,4
		His57Asp	0.04	NA	10	2,3,4

### Genetic variability

To investigate the specificity of genetic mutations in MTBC isolates from The Gambia, we compared missense mutation frequencies in Gambian isolates to those in the rest of West Africa and global datasets. Among over 100,000 mutations identified across 100 countries, 12,411 mutations in all drug resistance genes (11.6%) originated from The Gambia, underscoring the country’s notable contribution to the global mutation pool. Mutations in genes associated with drug resistance were found at significantly higher frequencies in The Gambia compared to global averages, particularly in key genes such *as rpo*B (RIF resistance), *inh*A (INH resistance), and *emb*B (EMB resistance). For example, the *rpo*B Thr350Ile uncertain significant mutation by the WHO catalogue was observed in 26.9% of Gambian isolates compared to 0.99% globally (Fig. 2A). The *emb*C Ala307Thr mutation of uncertain significance appeared in 15.7% of Gambian isolates but was rare globally (Fig. 2C). These mutations were steadily identified in MTBC isolates over the study period (Supp Fig. 4). In addition, mutations of uncertain significance associated with resistance to second-line drugs, such as moxifloxacin, were more common in The Gambia and West Africa than the global average. For example, *gyr*B Ala403Sep and *gyr*A Leu398Phe were both found at higher frequencies in The Gambia (34% and 29%) and West Africa (41% and 31%), respectively, compared to the global frequency (Supp Fig. 5). Some non-resistance mutations, such as c.−100 C > T, were uniquely prominent in The Gambia, often co-occurring with ethambutol resistance (Supp Fig. 6).

**Fig. 4:**
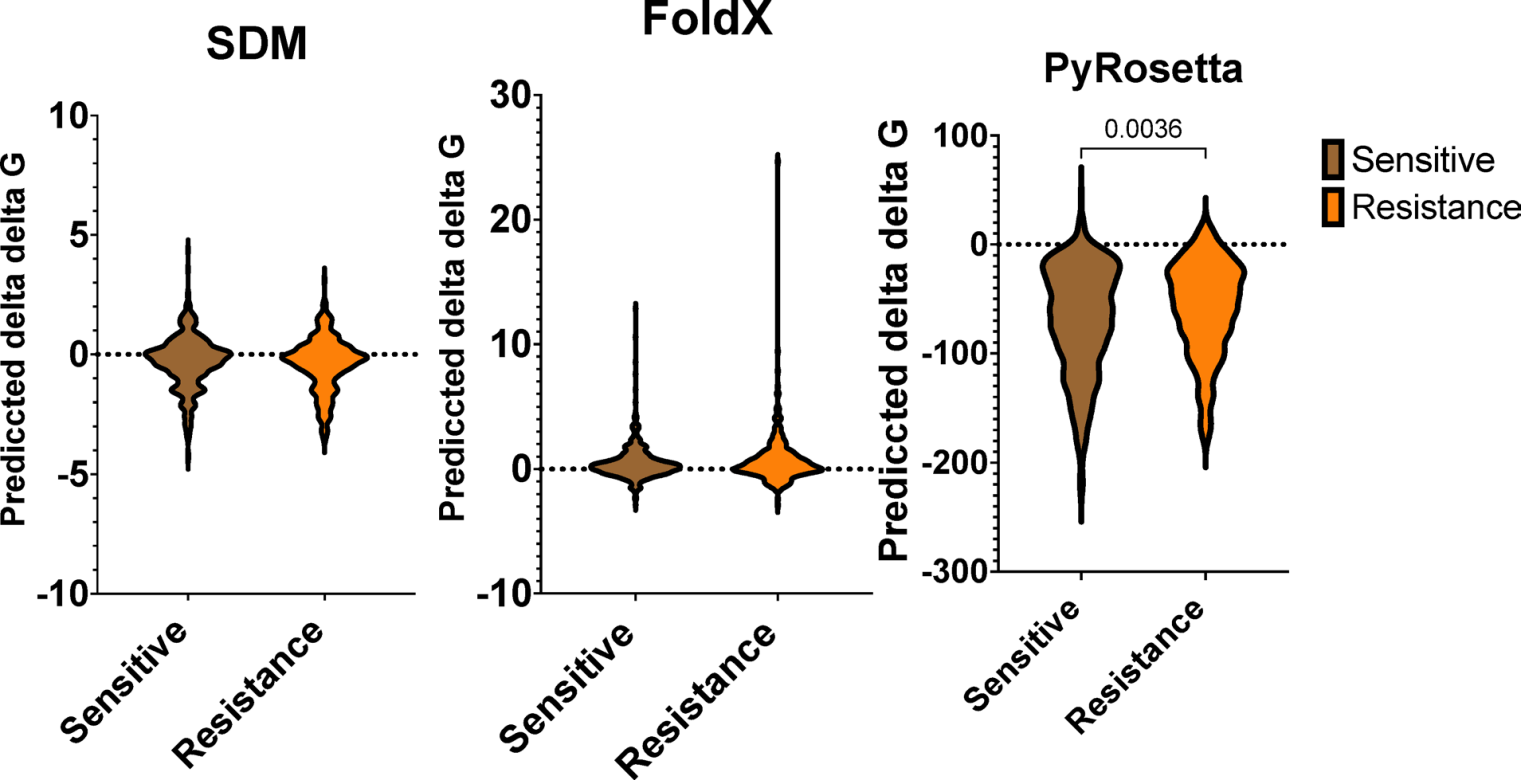
Predicted Delta Delta G (ΔΔG) of overall resistant and susceptible mutations in first-line drugs. Comparison of the distributions of ΔΔG for SDM (**A**), FoldX (**B**) and PyRosetta (**C**), which show different protein stability parameters between resistant and susceptible mutations.

**Fig. 5:**
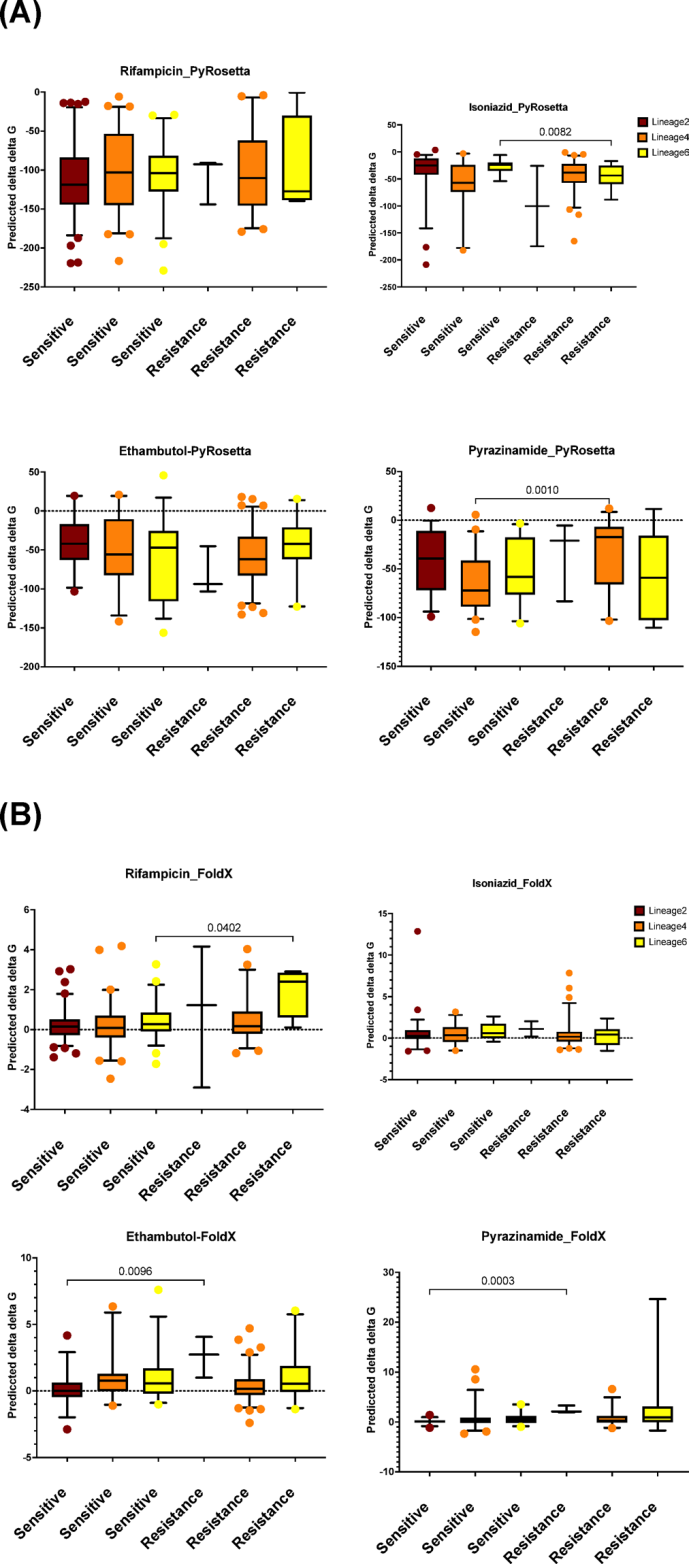
Predicted ΔΔG of resistant and susceptible mutations by first-line drug and MTBC lineages. Comparison of the distributions for (**A**) PyRosetta and (**B**) FoldX between resistant and susceptible mutations in lineage 2 (Brown), L4 (Orange) and L6 (Yellow).

### Structural and conservation properties of resistance and susceptible mutations

The distributions and structural properties of mutant sites were analysed based on residue depth, occluded surface packing (OSP), and relative solvent accessibility (RSA). A significant difference was observed between resistance and susceptibility-associated mutations across all structural property categories (two-tailed Mann-Whitney test, *p* < 0.05). It is interesting to note that resistance and susceptible mutations occur more frequently in tightly packed (OSP > 0.4) and less tightly packed (OSP < 0.4) regions of the protein structure, respectively (Fig. 3A). In terms of residue depth, susceptible mutations were observed more often at shallow residue depths (< 4 Å), while resistant mutations were concentrated at greater depths (8–9 Å) (Fig. 3B). Similar trends were also observed for RSA, where the resistance mutations were found at a higher frequency in solvent-inaccessible regions (RSA < 20%). In contrast, susceptible mutations were more frequently located in solvent-accessible regions (Fig. 3C).

Additionally, the conservation levels of resistance- and susceptibility-associated mutation alleles were analysed. Resistance mutations showed significantly greater conservation than background and susceptible mutations. Specifically, at conservation grades 5–9, the density of resistant mutations exceeded that of background and susceptible mutations. Conversely, at conservation grades 1–4, the density of susceptible mutations was higher than that of background and resistant mutations (Fig. 3D). Overall, resistant mutations showed greater conservation than susceptible mutations (one-tailed Wilcoxon matched-pairs signed-rank test, *p* < 0.05), highlighting their potential functional and evolutionary significance.

### Mutant stability prediction

The values of the ΔΔG (change in free energy) were analysed to predict the impact of resistant and susceptible mutations on protein stability. No significant differences in ΔΔG were observed between resistance and susceptible mutation using FoldX and SDM (Fig. 4). SDM predicted median ΔΔG values of −0.21 for resistance and − 0.18 for susceptible mutations. On the other hand, FoldX predicted median ΔΔG values of 0.31 for resistance and 0.24 for susceptible mutations. Both tools suggested that these mutations could severely destabilise or stabilise the protein (absolute ΔΔG > 0.5).

In contrast, PyRosetta analysis revealed significant differences in ΔΔG values (two-tailed Mann-Whitney test, *p* < 0.05). Resistant mutations showed a relatively more significant destabilising effect (median ΔΔG=−52.74) compared to susceptible mutations (median ΔΔG=−62.28) (Fig. 4).

To further investigate ΔΔG differences between resistant and susceptible mutations, mutations were grouped by first-line anti-tuberculosis drugs (RIF, INH, EMB, and PZA) and categorised by MTBC lineages. No significant differences between resistant and susceptible mutations were observed for any of the four drugs using SDM (Supp Fig. 7). In contrast, PyRosetta analysis revealed statistically significant differences (two-tailed Mann-Whitney test) for INH in Lineage 6 and EMB in Lineage 4 (Fig. 5A). FoldX analysis also identified substantial differences in ΔΔG values for RIF in Lineage 6 and EMB and PZA in Lineage 2 (Fig. 5B).

## Discussion

This study provides insights into the genomic landscape of *Mycobacterium tuberculosis* complex (MTBC) isolates in The Gambia over nearly two decades, shedding light on the country’s epidemiology, lineage diversity and drug resistance (DR) patterns. Consistent with prior studies in West Africa reviewed by de Jong et al. (2010), MTBC Lineage 4 (L4) and 6 (L6) were found to dominate, collectively accounting for 94% of TB cases.

The demographic trends observed are consistent with global TB patterns. Most cases were identified in male patients (72.5%), reflecting the higher incidence of TB among men worldwide^1^. The patient’s age distribution, with the highest burden among individuals aged 18–29 and 30–44, underscores the significant impact of TB on economically and socially critical population segments in The Gambia. These findings emphasise the importance of targeted interventions to mitigate the disease’s socioeconomic impact in The Gambia.

L4, the most prevalent lineage globally, accounted for 67.2% of isolates, likely due to its high virulence^37^, rapid progression to active TB^38^, and adaptability to diverse environments^6^. Within L4, the overrepresentation of sub-lineages L4.3 (LAM) and L4.1 further supports the role of these genetic clusters in driving their success in this population. LAM is a well-known lineage associated with increased virulence and transmission potential, which may explain its widespread distribution in this region^39^. In contrast, L6, which accounted for 26.6% of the isolates, remains mainly geographically restricted to West Africa. This highlights the importance of region-specific interventions that consider local strains’ genetic makeup^40^. The predominance of sub-lineage L6.1 highlights its evolutionary adaptation and persistence within this population, likely shaped by unique host population genetic susceptibility to this strain in the region^41^.

The detection of 19 multidrug-resistant (MDR) *Mycobacterium tuberculosis* complex isolates with a trend of growing number detected in recent years highlights an emerging public health concern for The Gambia and the need to expand treatment options with new shorter MDR-TB regimens. The markedly higher prevalence of isoniazid (INH) monoresistance (90 isolates) compared with rifampicin (RIF) monoresistance (10 isolates) suggests that INH resistance may be an emerging priority, as it is used in prophylaxis treatment guidelines^42^. The new, shorter rifapentine-based TB prophylaxis, rifapentine-isoniazid (3HP), for 3 months, may be suitable for addressing the growing INH monoresistance, which fuels MDR-TB.

We identified five top *rpo*B mutations of uncertain rifampicin resistance significance: Thr350Ile, c.−199 C > T, Ser450Leu, Asp435Val, and Ile770Val. Ser450Leu and Asp435Val, located within the rifampicin resistance-determining region (RRDR; codons 426–452), are detectable by GeneXpert *MTB/RIF* Ultra assays^43^. The others, located outside the RRDR escaping GeneXpert detection, have not been confirmed to confer resistance to RIF. Of note, Thr350Ile, which likely affects RIF binding to RNA polymerase^44^, was common in the *M. africanum* lineage 6 (26.9% in The Gambia; 50.9% in West Africa), suggesting its potential as a lineage-specific marker. Given its prevalence, phenotypic validation is a priority, and confirmation could inform the expansion of *GeneXpert* probe designs to improve rifampicin resistance detection in West Africa.

INH resistance was dominated by *kat*G Ser315Thr, which globally accounts for 90–95% of INH resistance, but occurred less frequently in Gambian isolates than in other regions^45^. The second most frequent variant, c.−497delA in the *kat*G promoter, may affect transcriptional regulation, though its phenotypic relevance remains unconfirmed, and was almost exclusive to Gambian lineage 4 (lineage 4.1.2.1) isolates, suggesting a local endemic cluster in circulation. Additional low-frequency variants included c.−777 C > T and c.−154G > A in the *inh*A promoter, both potentially regulatory but unvalidated mutations, and Pro44Arg in *ahp*C, a compensatory mutation without a direct resistance effect.

For ethambutol (EMB), Thr113Arg in *emb*A and Ala307Thr in *emb*C, both arabinosyltransferases, were enriched in lineage 6 but lack definitive resistance validation^46^. In contrast, the well-characterised Met306Val/Met306Ile mutations in *emb*B, responsible for 50–70% of EMB resistance, were less frequent in West Africa and absent in lineage 6, occurring mainly in lineages 2 and 4, indicating strong lineage specificity for this EMB resistance-associated mutation^47^. Pyrazinamide-associated variants included Pro766Leu in *clp*C1 of uncertain significance and three low-frequency *pnc*A mutations: Leu172Pro (C-terminal; impairs folding)^48^, Asp63Ala (near metal-binding site; linked to resistance), and His57Asp (active site; common in intrinsically resistant *M. bovis*)^49^. Although rare, these variants highlight the need for sustained PZA resistance surveillance, particularly in settings where phenotypic testing is technically challenging. The diversity of *pnc*A mutations underscores the urgency for rapid molecular diagnostics capable of detecting both canonical and rare resistance-associated variants^50^.

These findings emphasise the urgent need for continuous drug resistance (DR) surveillance and the revision of treatment protocols to reflect evolving local resistance patterns. The integration of molecular diagnostics into West African national TB programmes is crucial for the timely and accurate detection of DR strains. They will accelerate clinical decision-making and strengthen End TB control. Region-specific genomic surveillance is particularly important, as illustrated by the high frequency of locally enriched variants such as *rpo*B Thr350Ile and *emb*C Ala307Thr, which may be epidemiologically significant despite their uncertain role in resistance. Targeted laboratory investigations are needed to clarify the clinical and fitness impacts of these variants, especially those not yet confirmed in the WHO mutation catalogue.

Analysis of structural properties revealed key differences between resistant and susceptible mutations. Resistance-associated mutations were more frequently located in tightly packed regions of the protein structure (OSP > 0.4) and solvent-inaccessible regions (RSA < 20%), suggesting that they may disrupt proteins’ core structural stability^51^. In contrast, susceptibility-associated mutations were more often in less tightly packed, solvent-accessible regions. Additionally, resistance-associated mutations exhibited higher conservation grades (5–9) than susceptibility-associated and background mutations, indicating their critical role in maintaining essential protein functions. These findings align with the hypothesis that resistance-associated mutations often occur at functionally or structurally critical residues under strong evolutionary constraints^52^. Understanding these distinctions could inform drug design by prioritising highly conserved and structurally significant target sites.

The stability analysis demonstrated variations in the predictive capabilities of different computational tools. While SDM and FoldX did not identify significant differences in ΔΔG values between resistance and susceptibility-associated mutations, but PyRosetta did. Resistance-associated mutations showed a relatively greater destabilising effect (median ΔΔG = −52.74) than susceptibility-associated mutations (median ΔΔG = −62.28), suggesting that PyRosetta may be a more sensitive method. These destabilising effects may reflect structural alterations that disrupt drug-binding interactions or enable conformational changes, thereby reducing drug efficacy. Grouping mutations by first-line anti-TB drugs and MTBC lineages provided further resolution. For example, PyRosetta detected significant ΔΔG differences for INH-associated mutations in lineage 6 and EMB-associated mutations in lineage 4, while FoldX highlighted significant differences for RIF in lineage 6, and for EMB and PZA in lineage 2. These lineage-specific findings underscore the importance of considering genetic background and selective pressures when studying resistance mechanisms and designing treatment strategies.

While this study offers valuable insights into the genomic landscape of MTBC isolates in The Gambia, several limitations must be acknowledged. Although the dataset spans the period 2002–2021, sampling was temporally uneven, with most isolates collected between 2012 and 2015. This imbalance may limit the resolution of temporal analyses and the interpretation of long-term evolutionary trends. In addition, isolates were primarily obtained from the Greater Banjul Area and may not fully represent MTBC diversity and resistance patterns across rural regions of The Gambia. Whole-genome sequencing was conducted on cultured isolates, which may introduce culture-related bias, particularly for slow-growing strains such as *M. africanum* lineage 6. Culture-based approaches may also reduce the detection of mixed infections or within-host diversity due to selective bottlenecks during subculturing. Drug-resistance classification relied on established bioinformatic pipelines and the current WHO resistance catalogue, with greater emphasis on first-line drugs. As resistance interpretation frameworks continue to evolve, mutations presently classified as having “uncertain significance” may be redefined with additional phenotypic evidence. Moreover, phenotypic drug-susceptibility testing was not systematically available for all isolates, limiting comprehensive genotype–phenotype validation. Structural analyses and ΔΔG predictions were based on computational modelling approaches that remain limited^53^. While multiple tools were applied to increase robustness, in silico predictions do not directly confirm phenotypic resistance, protein fitness effects, or clinical impact. In particular, predicted structures and static models may not fully capture dynamic conformational changes or drug-bound states, and experimental validation remains necessary. Finally, this study did not incorporate transmission modelling, host genetic data, HIV stratification, or detailed analyses of treatment outcomes. Integration of genomic, clinical, and epidemiological data in future studies will further clarify the determinants of lineage distribution and resistance evolution. Despite these limitations, this study represents one of the largest genomic characterisations of MTBC isolates in West Africa and provides a comprehensive framework for understanding lineage-specific resistance patterns and their potential structural consequences.

## Conclusion

This genome-wide analysis of 1,803 *Mycobacterium tuberculosis* complex (MTBC) isolates provides a detailed overview of lineage distribution and drug-resistance variation in The Gambia. The predominance of globally distributed Lineage 4 alongside the West Africa–restricted Lineage 6 highlights the coexistence of internationally successful and regionally adapted MTBC populations. Genotypic profiling identified a considerable proportion of mutations classified as having uncertain resistance significance, particularly within lineage 6, including lineage-enriched mutations in *rpoB* and *embC* that are locally enriched compared to global datasets. These findings underscore the importance of region-specific interpretation of resistance-associated mutations. Structural analyses further showed that resistance-associated mutations tend to occur in conserved and structurally constrained regions of drug-target proteins, supporting their potential functional relevance. Although computational modelling suggested lineage- and drug-specific differences in mutation impact, experimental validation is required to confirm phenotypic consequences. Nonetheless, integrating large-scale genomic surveillance with structural bioinformatics provides additional context for understanding resistance mechanisms beyond catalogue-based classification. Overall, this study reinforces the value of sustained genomic surveillance integrated with functional analysis to refine resistance interpretation and support End Tuberculosis strategies in high-burden settings.

## Supplementary Information

Below is the link to the electronic supplementary material.

Supplementary Material 1

## Data Availability

The datasets generated and/or analysed during the current study are available in the European Nucleotide Archive (ENA) repository, with the Accession numbers PRJEB87376 and PRJEB53138.
