# Supplementary material for: Genome-wide analyses of Mycobacterium tuberculosis complex isolates reveal insights into circulating lineages and drug resistance mutations in The Gambia
Source: Sci Rep. 2026 Mar 4;16:12005. doi: 10.1038/s41598-026-42003-2 (PMC13068966; doi:10.1038/s41598-026-42003-2)
Supplement: Supplementary file 1 — Supplementary Material 1 [file 41598_2026_42003_MOESM1_ESM.docx]

**Supplementary figures**


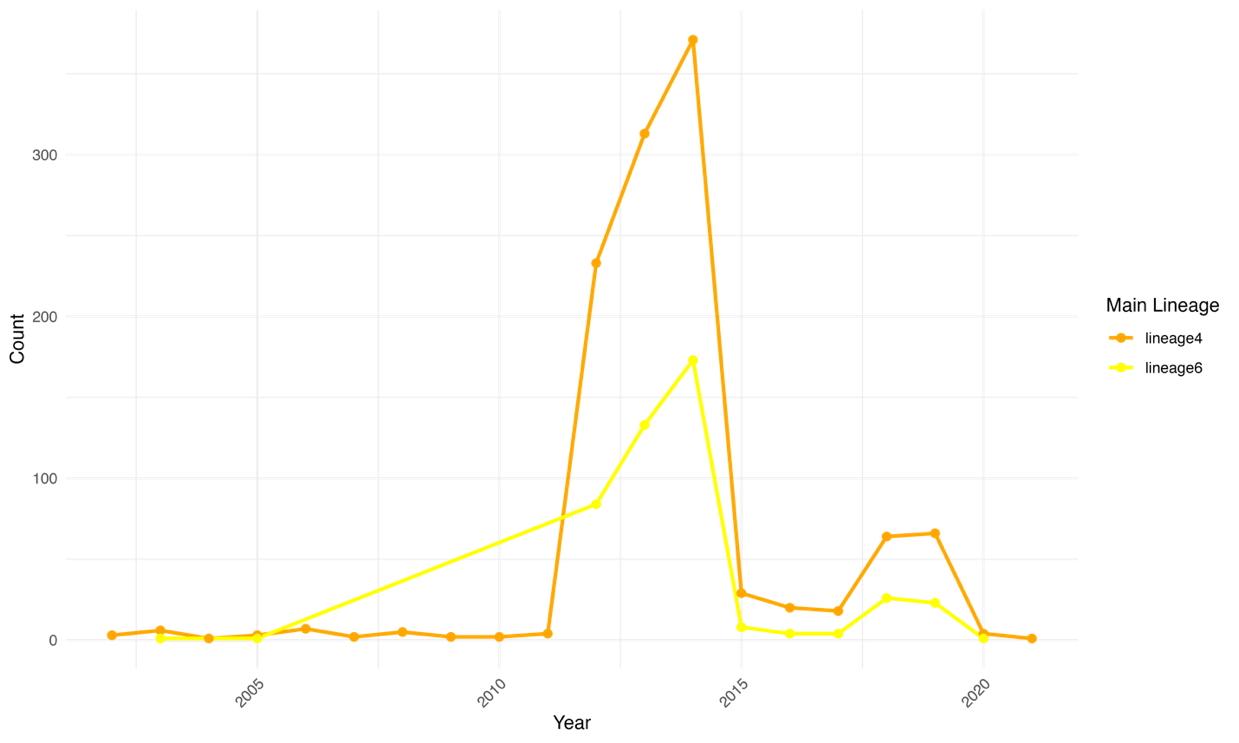


**Supp Figure 1: Distribution of MTBC lineage 6 and 4 from 2002 to 2021.**

A


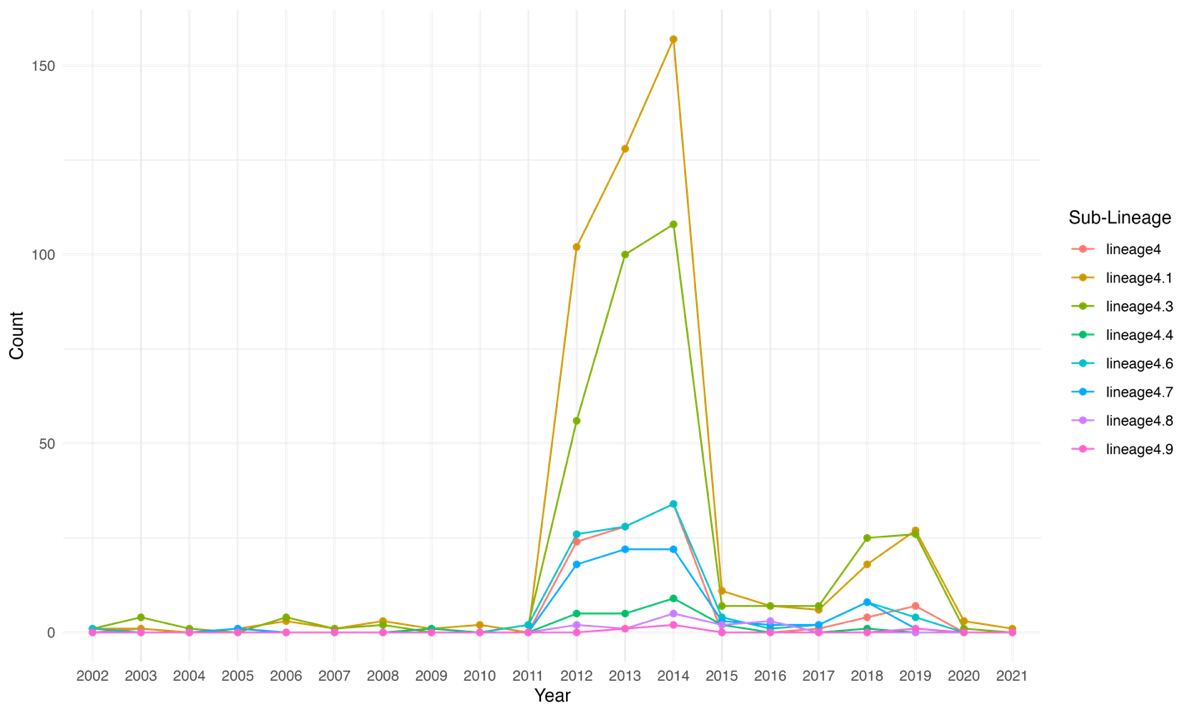


B


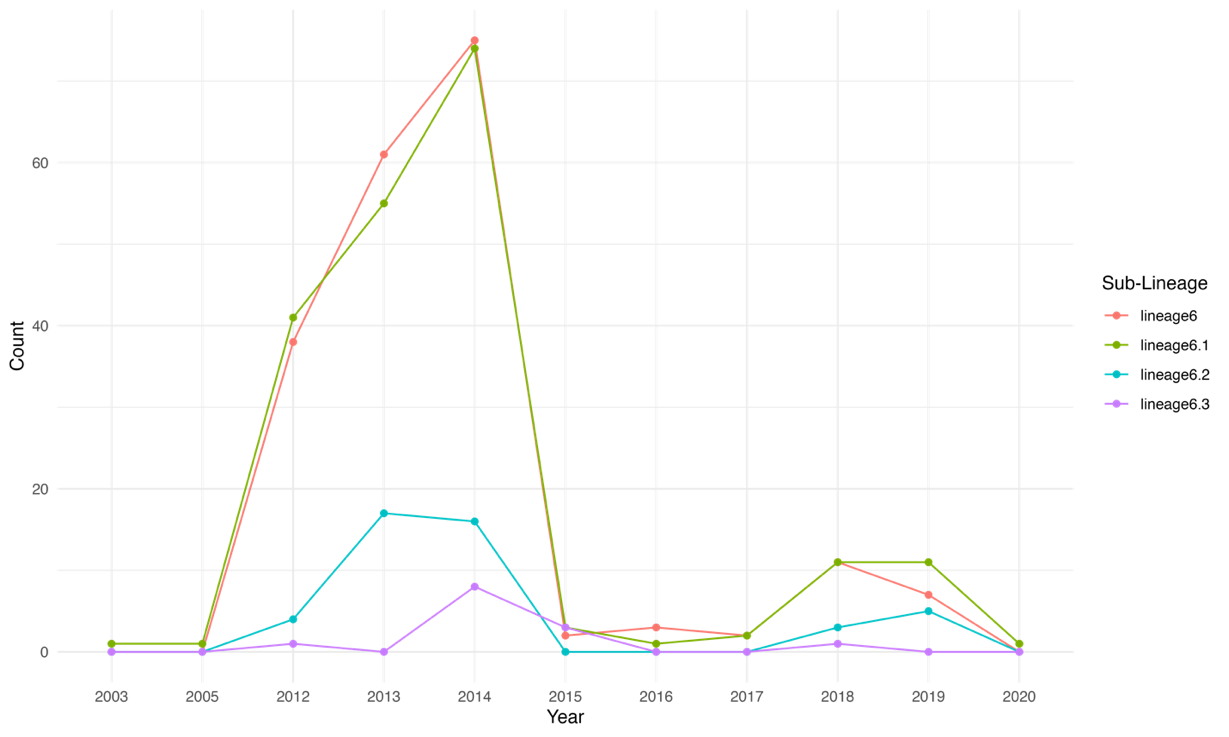


**Supp Figure 2: Distribution of MTBC sub-lineages from 2002 to 2020. Panel** (A) is for MTBC lineage 4 and (B) for MTBC lineage 6.


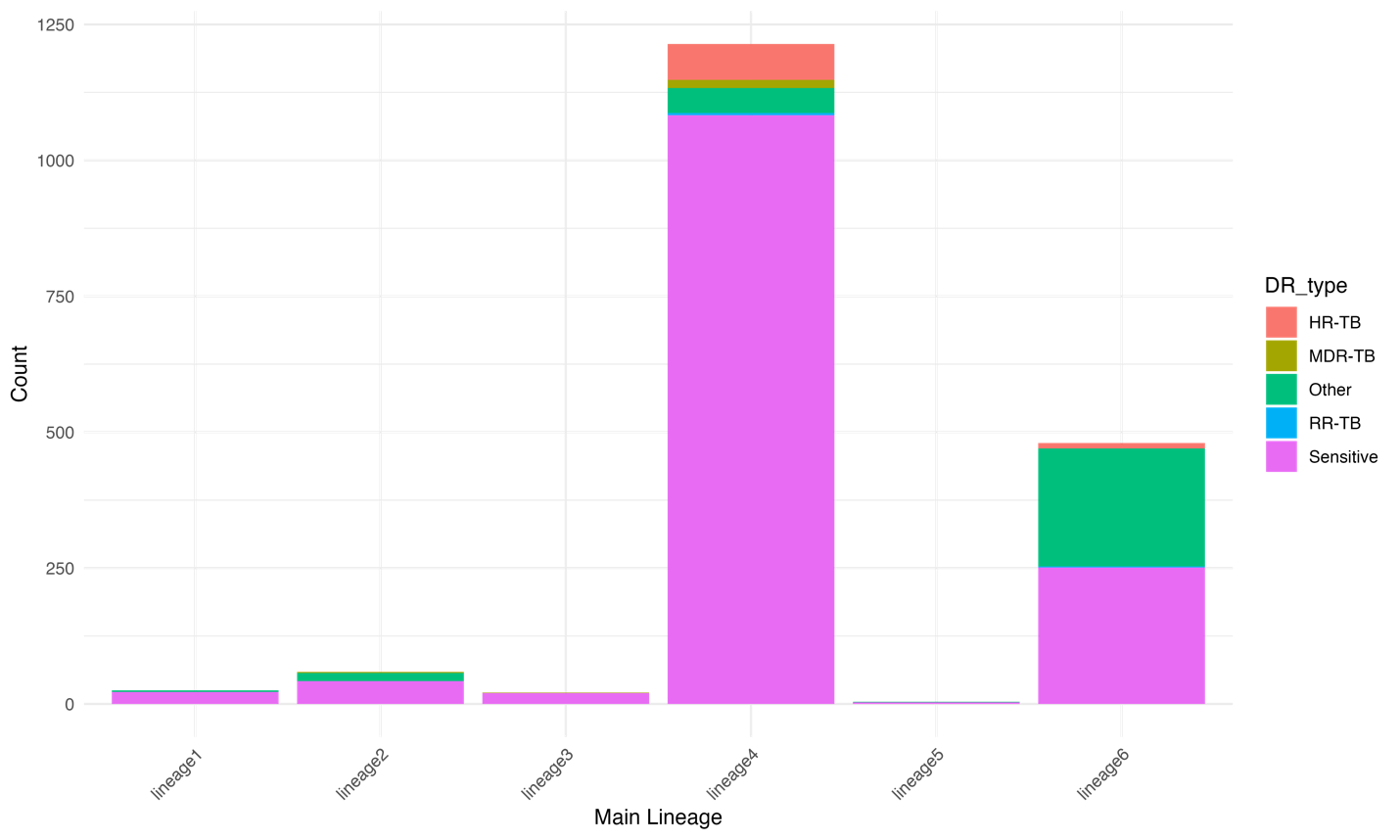


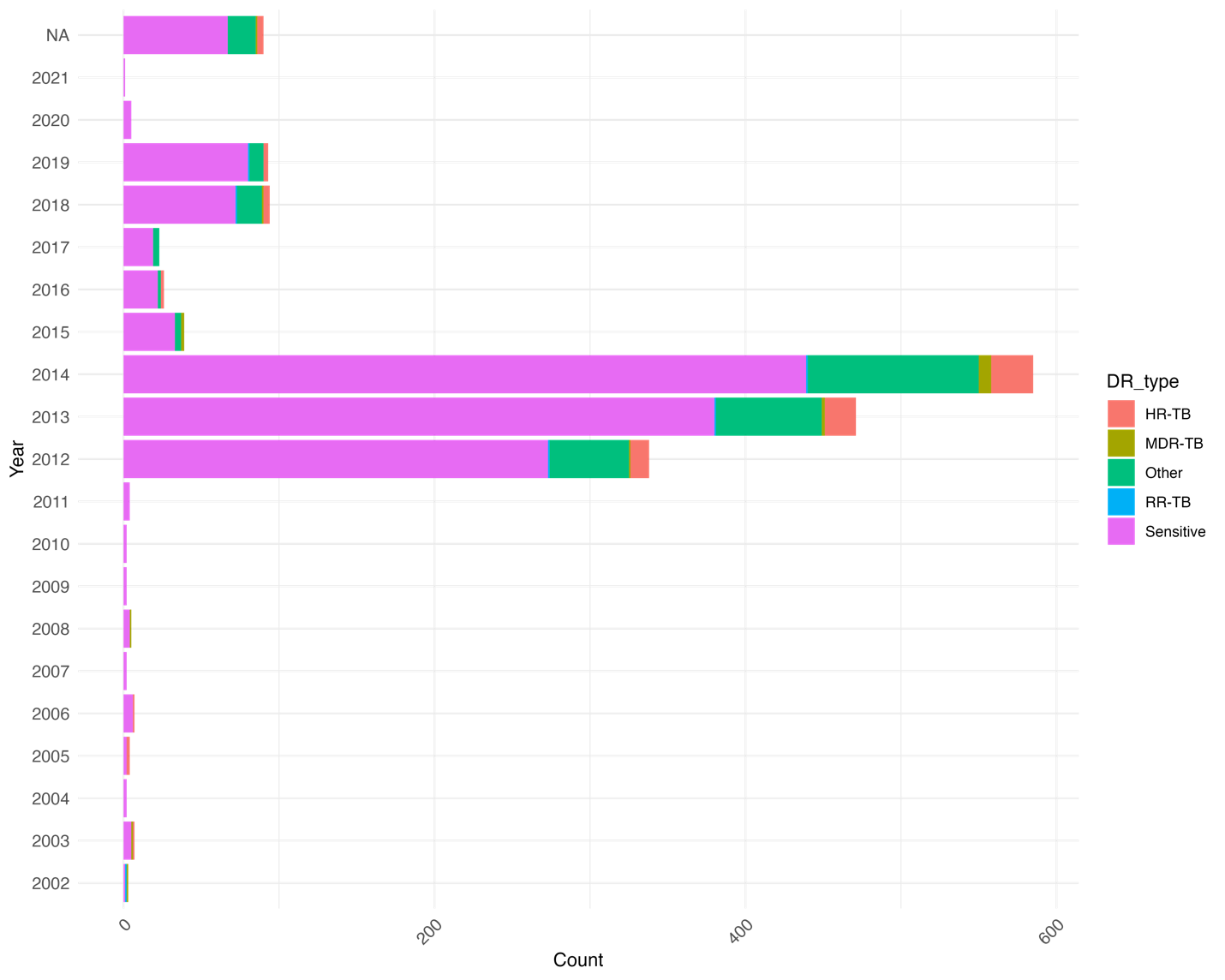


**Supp Figure 3: Distribution of drug-resistant strains across MTBC lineages (A) and over time (B) in The Gambia.**


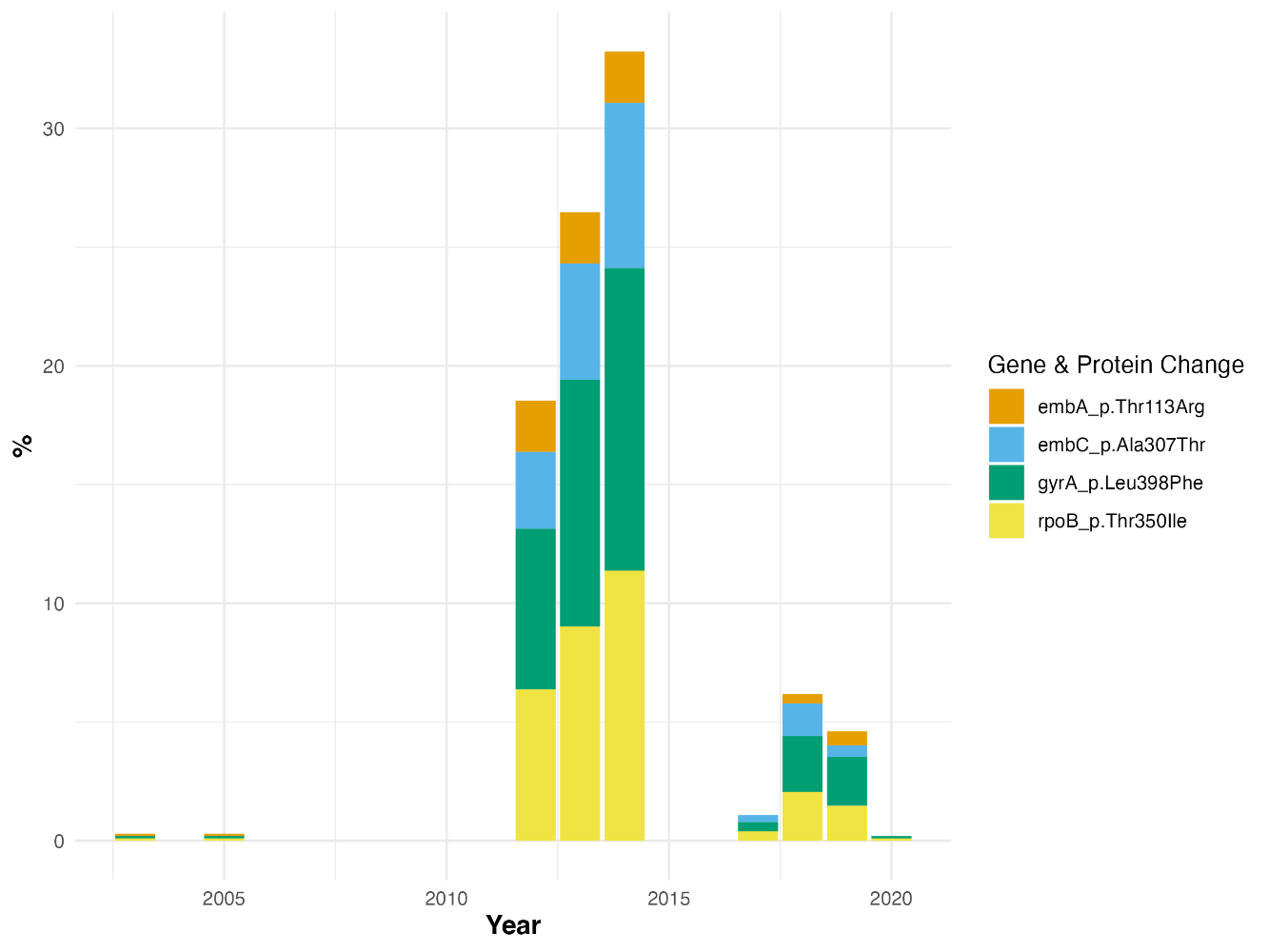


**Supp Figure 4: Prevalence of mutations of uncertain resistance significance over time**

**
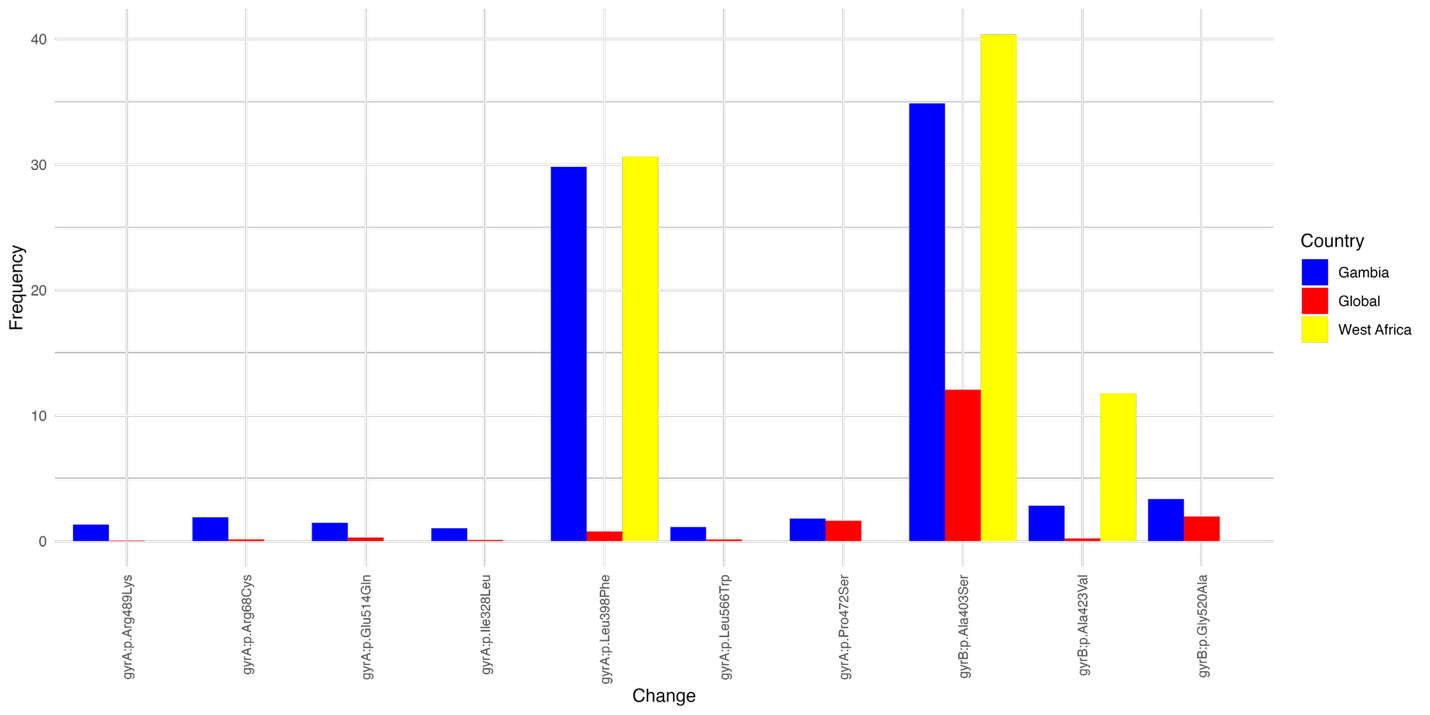
**

**Supp Figure 5: The prevalence of mutations with uncertain significant resistance according to the WHO catalogue in moxifloxacin drug in The Gambia, West Africa and the global database.**

**
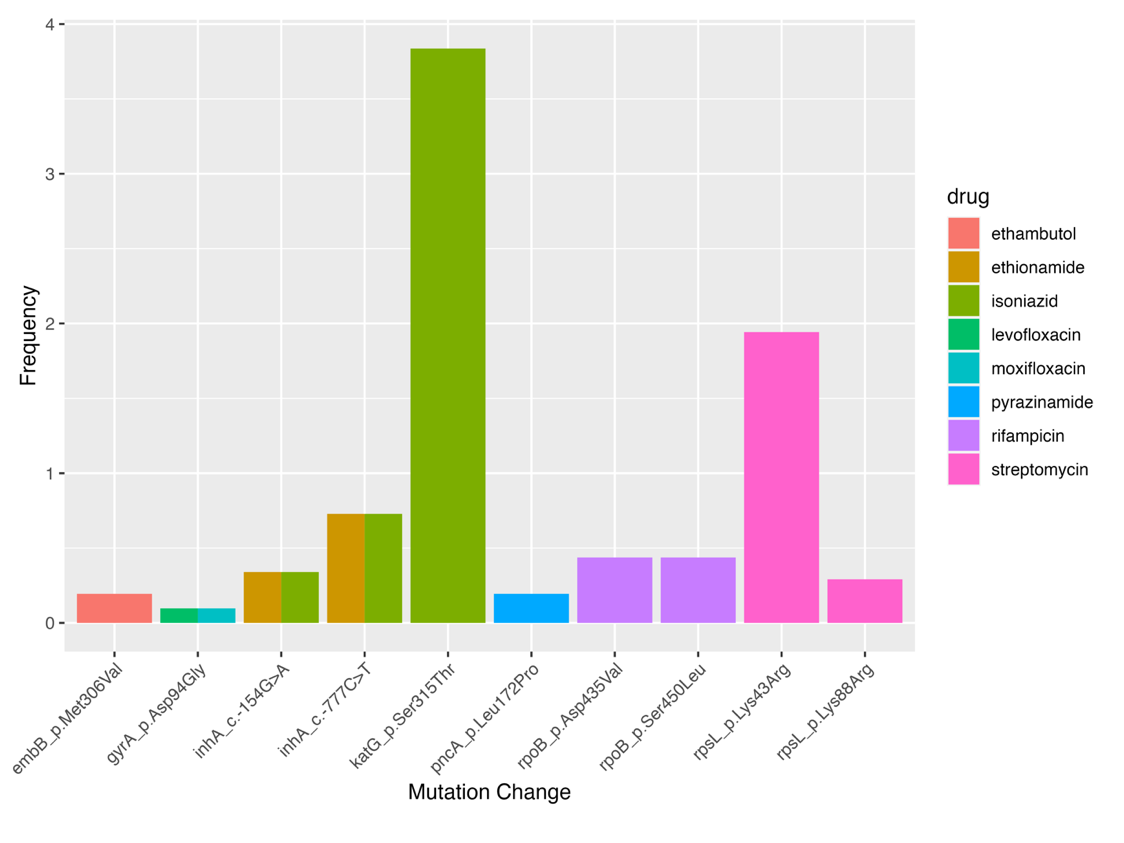
**

**B.**

**
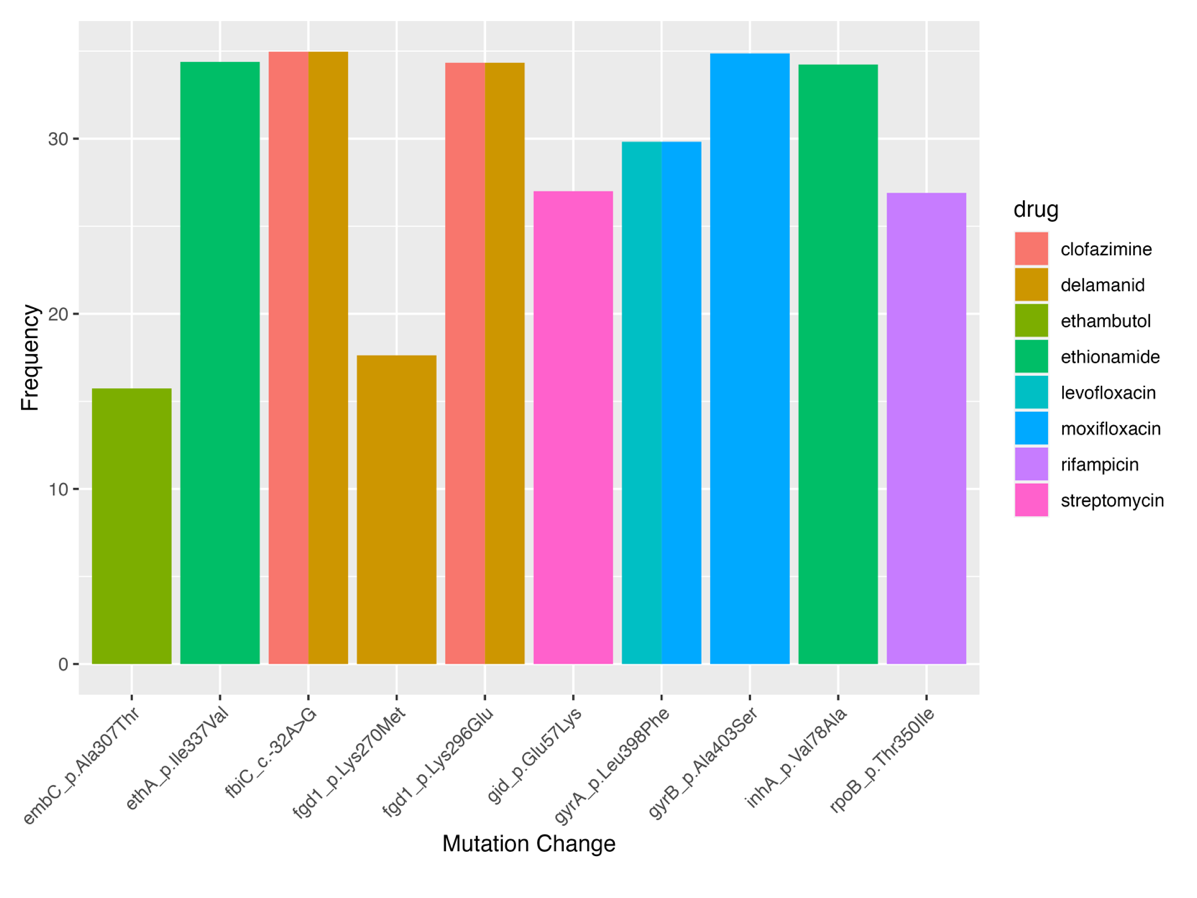
**

**C.**

**
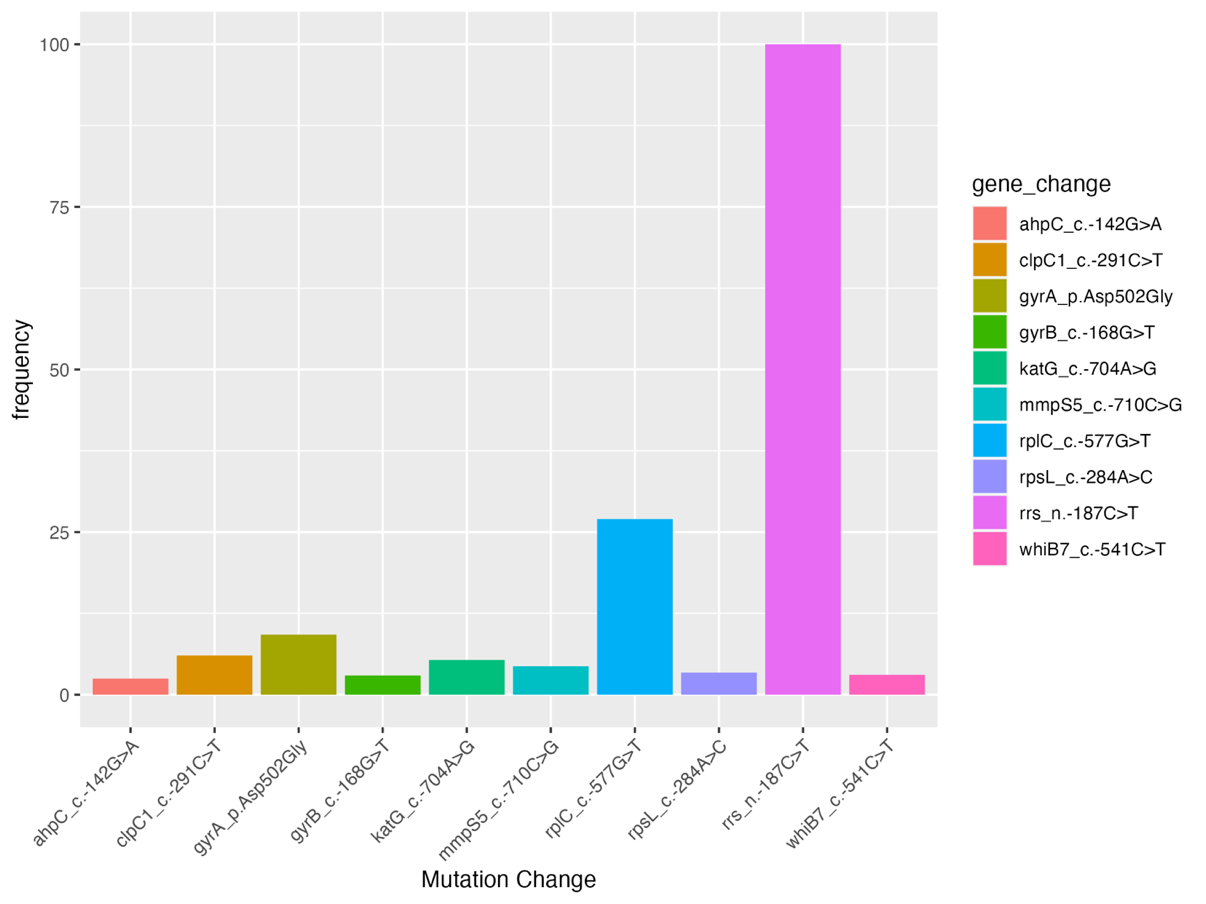
**

**Supp Figure 6: The distribution of variants across different categories, highlighting their association with specific genes or drugs in the Gambian isolates.** The categories are classified according to the WHO catalogue: (A) associated with resistance, (B) of uncertain significance, and (C) not in the WHO catalogue.

**Supp Figure 7: SDM predicted delta delta G of resistant and susceptible mutations per MTBC lineages for the first-line drugs**
